# Molecular profiling of TOX-deficient neoplastic cells in cutaneous T cell lymphoma

**DOI:** 10.1007/s00403-019-02000-0

**Published:** 2019-11-01

**Authors:** Jingkai Xu, He Huang, Shangshang Wang, Yanzhen Chen, Xueli Yin, Xuejun Zhang, Yaohua Zhang

**Affiliations:** 1 Institute of Dermatology, Huashan Hospital, Fudan University, 12 Wulumuqi Zhong Road, Jing’an District, Shanghai, 200040 China; 2 Department of Dermatology, The First Affiliated Hospital, Anhui Medical University, 81 Meishan Road, Hefei, 230032 China; 3 Key Laboratory of Dermatology, Ministry of Education, Anhui Medical University, Hefei, 230032 China; 4 Worldwide Medical Center, Huashan Hospital, Fudan University, 12 Wulumuqi Zhong Road, Jing’an District, Shanghai, 200040 China; 5 Department of Hematology, Huashan Hospital, Fudan University, 12 Wulumuqi Zhong Road, Jing’an District, Shanghai, 200040 China

**Keywords:** Cutaneous T cell lymphoma, TOX, RNA sequencing analysis, Differentially expressed gene, Signaling pathway

## Abstract

Cutaneous T cell lymphoma (CTCL) is a rare but potentially devastating primary cutaneous lymphoma. CTCL is characterized by localization of neoplastic T lymphocytes to the skin, with mycosis fungoides (MF) and its leukemic form, Sézary syndrome (SS) being the most common variants. Thymocyte selection-associated high-mobility group box (TOX) gene has been found to be highly expressed in MF and SS. It is reported that higher expression levels of TOX in patients will increase risks of disease progression and poor prognosis. However, the molecular events leading to these abnormalities have not been well understood. To better understand the molecular mechanism underlying TOX-mediated differentially expressed genes (DEGs) in CTCL, and to identify DEGs pathways triggered after knockdown of TOX gene in the CTCL cell line Hut78, we employed two shRNA-mediated lentiviruses to knock down TOX gene in the skin lymphoma cell line HuT78. RNA sequencing (RNAseq) analysis was applied to analyze DEGs, DEGs GO and their corresponding pathways. Knockdown of TOX can induce upregulation of 547 genes and downregulation of 649 genes, respectively. HOXC9 was the most significant downregulated gene. Most DEGs are enriched in malignancies and relate to the Wnt and mTOR signaling pathways, and therefore they can regulate cellular processes and induce different biological regulation. Transcriptome analysis of DEGs after knockdown of TOX in our study provides insights into the mechanism of TOX in CTCL and suggests candidate targets for therapy of CTCL.

**Electronic supplementary material:**

The online version of this article (10.1007/s00403-019-02000-0) contains supplementary material, which is available to authorized users.

## Introduction

CTCLs are a heterogeneous group of non-Hodgkin lymphoproliferative disorders characterized by accumulation and expansion of neoplastic T lymphocytes to the skin [28]. MF and SS constitute two main subtypes of CTCL. While MF primarily affects the skin, SS is characterized by the presence of circulating malignant Sézary cells. Together, MF and SS account for 65–80% of CTCL cases [9, 11, 23]. Although accumulative evidence indicates that defects in apoptosis and cell cycle control are critical in disease pathogenesis [5, 21], the molecular mechanism leading to these abnormalities has not been well understood yet.

The TOX gene was firstly described in 2002 [27], as a part of the superfamily of high-mobility group box proteins that act as regulators of gene expression, mainly by modifying the chromatin structure [10, 27]. TOX mRNA is most abundant in the thymus, liver and brain [27]. TOX is involved in lymphocyte maturation, and Zhang et al. demonstrated that TOX was highly and specially expressed in early MF [31]. After this, several studies have confirmed that TOX is aberrantly expressed in CD4^+^ /CD8^−^ neoplastic T cells in MF and SS [2, 6, 7, 15, 19, 20, 29], so as to be aberrantly expressed in CTCL with CD4^−^/CD8^+^ and CD4^−^/CD8^−^ phenotypes [24], differentiating malignant from non-malignant skin-infiltrating T cells found in benign inflammatory dermatoses [31]. Aberrant expression of TOX plays a central role in malignant survival, proliferation, and tumor formation in CTCL [7]. Stable knockdown of TOX in CTCL cells has promoted apoptosis and reduced cell cycle progression, leading to less cell viability and colony-forming ability in vitro and reducing tumor growth in vivo [7].

It is generally believed that abnormal gene expression is a key process in disease initiation and progression. Hut78 cell line derived from SS exhibits high expression of TOX, and TOX-deficient Hut78 cells can promote apoptosis and reduce cell cycle [7], but its mechanism is not very clear. Herein, we applied RNAseq analysis to further explore transcriptional changes including expressed genes (DEGs), DEG Gene Ontology (GO) and pathways in TOX-deficient Hut78 cells.

## Material and methods

### Cell culture

Human CTCL cell line Hut78 (ATCC no. TIB161) was cultured in RPMI 1640 and 10%FBS as described by the American Type Culture Collection (Manassas, VA). Infected CTCL cells were cultured in the above medium plus puromycin.

### Lentivirus infection

Lentivirus vector (hU6-MCS-Ubiquitin-EGFP-IRES-puromycin) and shRNA sequence were designed and synthesized by Genechem (Shanghai, China). Destination cells were infected with lentiviral supernatants, using 8 mg/ml polybrene and high virus titer for MOI ≥ 100. After 48–72 h of incubation, the supernatant was replaced by a medium containing 1 mg/ml puromycin.

### RNA isolation and quantitative real-time PCR (qRT-PCR)

Total RNA was isolated from cell pellets using TRIzol (Invitrogen, Thermo Fisher) according to the manufacturer’s protocol. cDNA synthesis was performed using the GoScript™ Reverse Transcription System Kit (A5000) from Promega. qPCR reactions were performed with FastStart Universal SYBR Green Master (Rox) from Roche. The experiments were performed according to the manufacturer’s instructions. The sequences of the primers used for qRT-PCR analyses are listed in Table S3. All reactions were run in triplicate. The CT values were calculated using the standard curve method.

### Western blotting

After lentivirus infection, HuT78 cell pellets were prepared by centrifugation at 300*g*, and then total cells were lysed in RIPA buffer (50 mM Tris, 150 mM sodium chloride, 1% Triton X-100, 0.1% sodium dodecyl sulfate, and 1% sodium deoxycholate). After removing insoluble material by centrifugation at 10,000*g* at 4 ℃ for 5 min, total protein concentration was determined using BCA assay as per manufacturer’s instruction with a microplate reader. 40 μg protein was used for SDS-PAGE gel electrophoresis (Bio-Rad) and transferred onto PVDF membranes (Bio-Rad). Blocking was done with 5% milk and then the membranes were incubated with primary antibodies, anti-TOX (1:1000 HPA018322, Sigma-Aldrich) or anti-actin (1:5000, A1978, Sigma-Aldrich) overnight at 4 ℃. After washing, membranes were incubated with secondary antibodies (peroxidase-conjugated, suitable for each primary antibody) for 2 h at room temperature. The signal was detected using Bio-Rad ChemiDoc XRS + System after adding Super Signal West Pico chemiluminescence.

### Apoptosis detection

The treated Hut78 cells (1 × 10^6^) using shRNA 1 construct were transferred to a 15 ml centrifuge tube. Annexin V binding buffer was added. After centrifugation at 2000 rpm for 5 min at 4 °C, the cells were washed three times and 100 µl of binding buffer, 5 µl of Annexin V-APC and 10 µl of 7-AAD stain (Thermo Fisher Scientific, Inc) were added and incubated in the dark for 25 min. Detection of apoptotic cells was performed by flow cytometry.

### Cell cycle analysis

The treated Hut78 cells (1 × 10^6^) using shRNA 1 construct were collected and fixed with 75% ice-cold ethanol at 4 °C overnight and then stained with 5 µl propidium iodide (Thermo Fisher Scientific, Inc.) at room temperature for 5 min in the dark. The cell cycle distribution was analyzed by flow cytometry.

### RNAseq analysis

Total RNA from infected cells was harvested and extracted by using TRIzol (Invitrogen, Thermo Fisher). Agilent 2100 Bioanalyzer (Agilent RNA 6000 Nano Kit) was used to perform quality control of the total RNA samples:RNA concentration, RIN value, 28S/18S and the fragment length distribution. mRNAs were isolated from total RNA with the oligo(dT) method. The mRNAs were fragmented and then first-strand cDNA and second-strand cDNA were synthesized. cDNA fragments were purified and resolved with EB buffer for end reparation and single nucleotide A (adenine) addition. cDNA fragments were next linked with adapters. Those cDNA fragments with suitable size were selected for the PCR amplification. Agilent 2100 Bioanalyzer and ABI StepOnePlus Real-Time PCR System were used in quantification and qualification of those libraries. Equimolar pooling of libraries was performed based on qPCR values and loaded onto an Illumina Hiseq platform (BGI, China).

## Results

### Genetic silencing of Tox in Hut78 cells

**Fig. 1:**
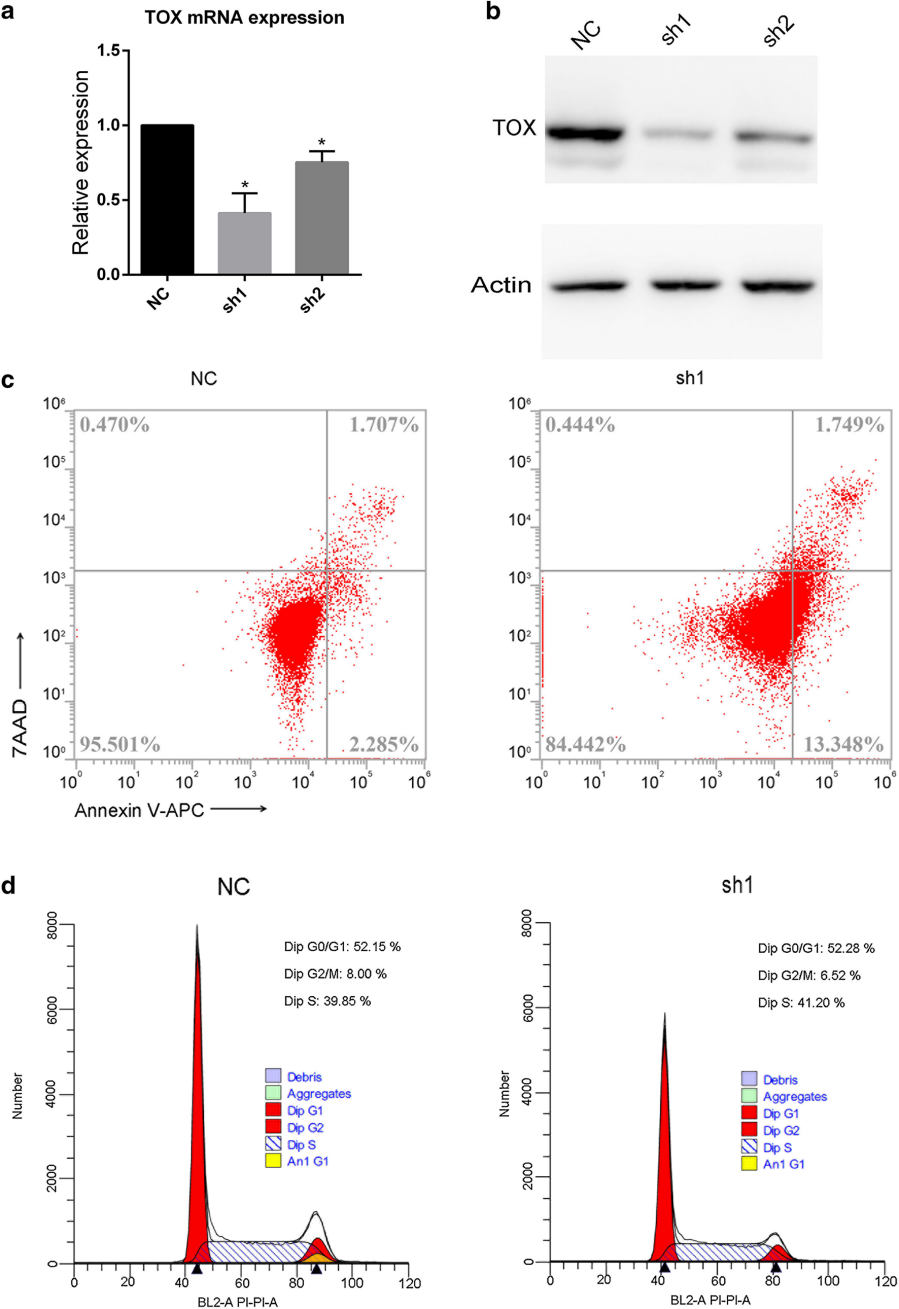
Lentivirus infection knockdown TOX gene expression. TOX knockdown by 2 shRNAs (sh1 and sh2, both specific for TOX mRNA) and negative control (a non-targeting shRNA). Infected cells were selected by puromycin (1 mg/mL) for 5 days. mRNA and protein were extracted for further analysis. **a** RT-qPCR was performed between group NC, sh1 and sh2, and primer qGAPDH and TOX were used. TOX was significantly reduced in sh1 (*p* value = 0.0114, *R*^2^ = 0.8305) and sh2 (*p* value = 0.0286, *R*^2^ = 0.7371). **b** Western blotting was performed with antibodies against TOX and actin proteins. *p < 0.05 by two-tailed Student’s *t* test with Welch correction. Error bars indicate standard error of the mean. Data shown here are representative of at least three independent experiments. **c** Annexin V-APC/7AAD flow cytometry assay showed that apoptotic cells were increased after knockdown of TOX. **d** Annexin/PI flow cytometry assay showed that more cells in the G0/G1 phase and less cells in the G2/M phase after knockdown of TOX

To investigate the transcriptional changes after TOX knockdown, two lentivirus targets were designed to knock down TOX gene in Hut78 cell line, as presented in Table S2. After lentivirus infection, RT-qPCR and Western blot were completed. TOX expression was significantly reduced in mRNA level as shown in Fig. 1a: Compared to the NC group, both sh1 and sh2 groups demonstrate significantly reduced TOX mRNA expression (*p < *0.05). TOX protein expression was also diminished as shown in Fig. 1b with the sh1 group showing more inhibition of TOX expression than the sh2 group. Annexin V-APC/7AAD flow cytometry assay was employed to analyze cell apoptosis, and we observed that apoptotic cells were increased after knockdown of TOX as shown in Fig. 1c. The cell cycle distribution analysis showed more cells in G0/G1 phase and less cells in G2/M phase after knockdown of TOX as shown in Fig. 1d.

### DEGs after TOX knockdown

After RNAseq and reads filtering, we mapped clean reads to reference genome by using Bowtie 2 [12] and then calculated the gene expression level for each sample with RSEM [13], a software package for estimating gene and isoform expression levels from RNAseq data. Subsequently, we calculated Pearson correlation between all samples by using cor, performed hierarchical clustering between all samples by using hclust, performed PCA analysis with all samples using princomp, and drew the diagrams with ggplot2 with functions of R. The number of genes and transcripts in each sample are shown in Table1. We further calculated the heat map of Pearson correlation among all samples, shown in Fig. S1a. Based on the expression information, we performed box plot analysis to show the distribution of the gene expression level of each sample, so that we could observe the dispersion of the distribution (results as shown in Fig. S1b). Based on the gene expression level, we could identify the DEGs between samples or groups. MA plots were used to show the distributions of DEGs in Fig. S1. Compared to the NC group, 3897 genes were overexpressed and 2702 genes were underexpressed in group sh1 (Fig. S1c). Compared to the NC group, 2723 genes were overexpressed and 3224 genes were underexpressed in group sh2 (Fig. S1d). Taken together, after TOX knockdown, a total of 547 genes were upregulated and 649 genes were downregulated. The top 20 downregulated genes are listed in Table 2 and the top 20 upregulated genes are listed in Table S1. Interestingly, we found that multiple genes in the HOX gene family were downregulated in TOX-deficient Hut78 cells.

**Table 1:** Genes and transcripts statistics

Sample	Total gene number	Known gene number	Novel gene number	Total transcript number	Known transcript number	Novel transcript number
NC_1	16,143	13,262	2881	28,211	14,795	13,416
NC_2	15,472	12,807	2665	25,537	13,585	11,952
NC_3	15,468	12,863	2605	25,801	13,699	12,102
sh1_1	15,089	12,530	2559	24,071	12,629	11,442
sh1_2	14,639	12,088	2551	21,614	11,028	10,586
sh1_3	15,023	12,387	2636	23,159	11,918	11,241
sh2_1	16,040	13,100	2940	28,513	15,031	13,482
sh2_2	16,281	13,395	2886	30,287	16,217	14,070
sh2_3	13,278	11,548	1730	20,495	11,131	9364

*Sample* sample name, *total gene number* the amount of all genes, *known gene number* the amounts of known genes, *novel gene number* the amounts of novel genes, *total transcript number* the amount of all transcripts, *known transcript number* the amounts of known transcripts, *novel transcript number* the amounts of novel transcripts

**Table 2:** Top 20 significantly downregulated genes after TOX knockdown

Gene name	GeneID	Length^a^	NC-expression^b^	sh1-expression	log2 Ratio (sh1/NC)^c^	*q* value^d^	*p* value^e^	sh2-expression	log2 Ratio (sh2/NC)	*q* value	*p* value
HOXC9	3225	1528	90,574	16,021	− 1.837919802	0	0	16,747	− 2.788427035	0	0
PIGS	94005	2643	54,217	7133	− 2.264951439	0	0	9093	− 2.929149771	0	0
TXNDC16	57544	2854	3017	68	− 4.810223171	0	0	313	− 3.622112876	0	0
GPATCH4	54865	2158	8047	2539	− 1.002977669	6.4747E-244	7.0734E-246	4285	− 1.26238857	0	0
CKB	1152	1475	3529	984	− 1.18131624	6.1571E-140	1.7733E-141	907	− 2.313317716	0	0
UNC119B	84747	4423	3865	1210	− 1.014248397	7.3431E-120	2.5525E-121	2313	− 1.09393589	4.2E-189	2.3E-190
ANP32E	81611	3446	1085.54	125.58	− 2.450521296	1.8879E-119	6.6251E-121	207.8	− 2.73837807	3.3E-188	1.8E-189
GBA	2629	2637	795.64	60	− 3.067868433	7.1629E-111	2.7981E-112	422.83	− 1.26527081	3.31E-50	8.35E-51
HOXC5	3222	1645	592	1	− 8.548240396	5.00096E-96	2.45025E-97	18	− 5.392761106	7.7E-159	4.9E-160
HOXC4	3221	1689	1196	281	− 1.428362385	2.4963E-64	2.18996E-65	323	− 2.241844062	3E-165	1.9E-166
PRR5-ARHGAP8	553158	1179	342.35	11.31	− 4.258588209	1.93483E-61	1.81269E-62	97.19	− 2.169824969	1.43E-46	4.03E-47
POR	5447	2509	860.3	170.31	− 1.675463721	1.45546E-58	1.43585E-59	216.96	− 2.340643496	1.6E-125	1.3E-126
MTPN	136319	3900	314.21	0	− 8.634372317	3.90708E-51	4.87628E-52	0	− 9.648818028	1.19E-62	2.22E-63
PMF1	11243	1122	1023.19	273.66	− 1.241404619	7.84823E-45	1.21854E-45	641.82	− 1.02606616	2.8E-46	7.97E-47
CDRT4	284040	2515	243.61	0	− 8.267216577	1.33826E-41	2.39238E-42	0	− 9.281662288	2.29E-51	5.62E-52
SDSL	113675	1311	325	36	− 2.513157937	7.66449E-38	1.61629E-38	40	− 3.375600555	1.42E-69	2.35E-70
CDKN2A	1029	1218	1132	351	− 1.028118053	6.07066E-37	1.30831E-37	191	− 2.920462157	2E-209	9.4E-211
PCSK1N	27344	1071	855	238	− 1.183749877	3.87281E-35	9.09008E-36	178	− 2.617279921	1.5E-141	1.1E-142
SURF1	6834	1046	189.76	0	− 7.906819136	6.41566E-34	1.60992E-34	0	− 8.921264847	3.28E-42	1.05E-42
ZNF616	90317	4386	295.55	35.94	− 2.378526937	1.48496E-32	4.00162E-33	18	− 4.390566151	3.72E-75	5.49E-76

^a^Gene length; ^b^gene expression of group NC; ^c^log2 transformed fold change between NC group and sh1 group; ^d^adjusted *p* value; ^e^*p* value

### GO analysis of DEGs

**Fig. 2:**
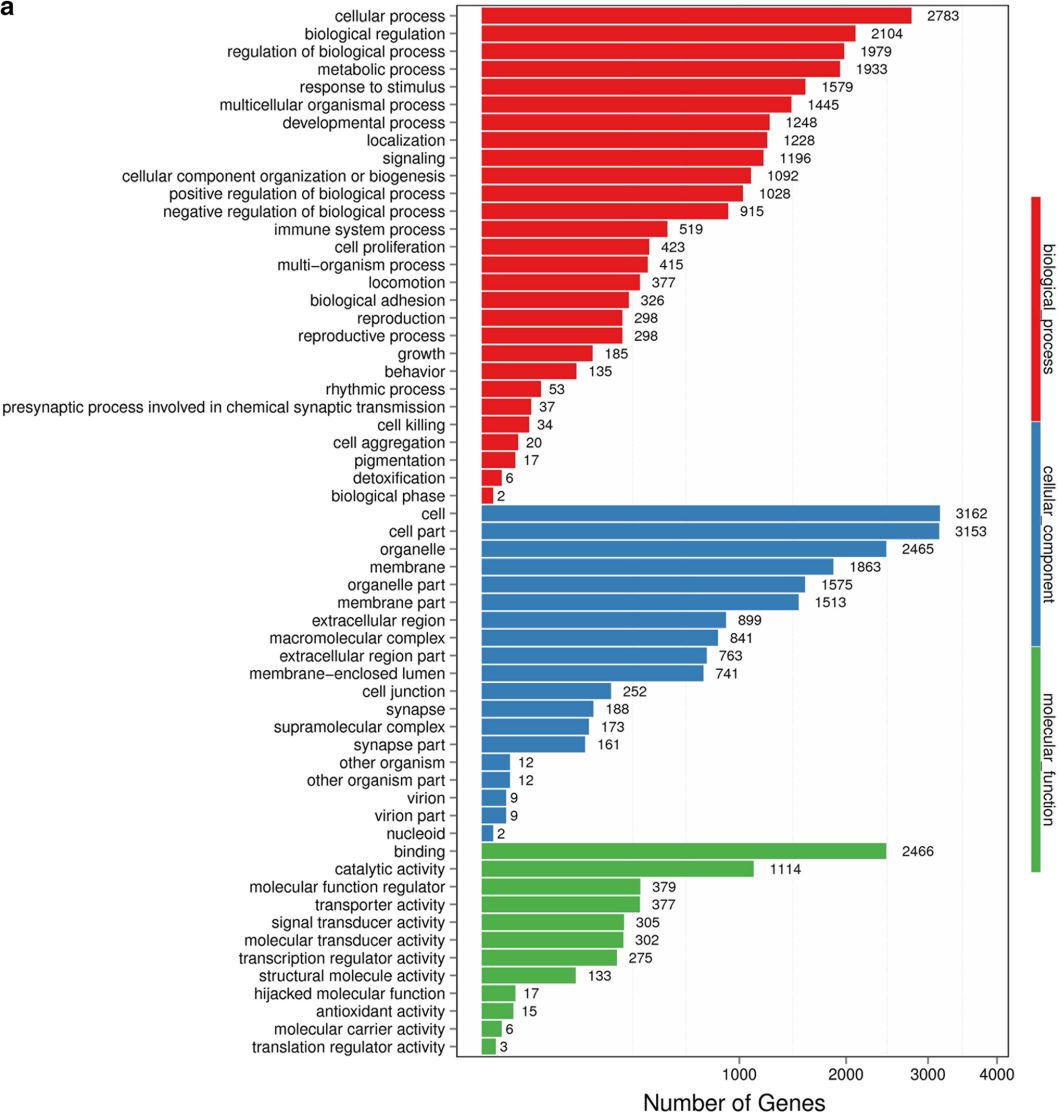

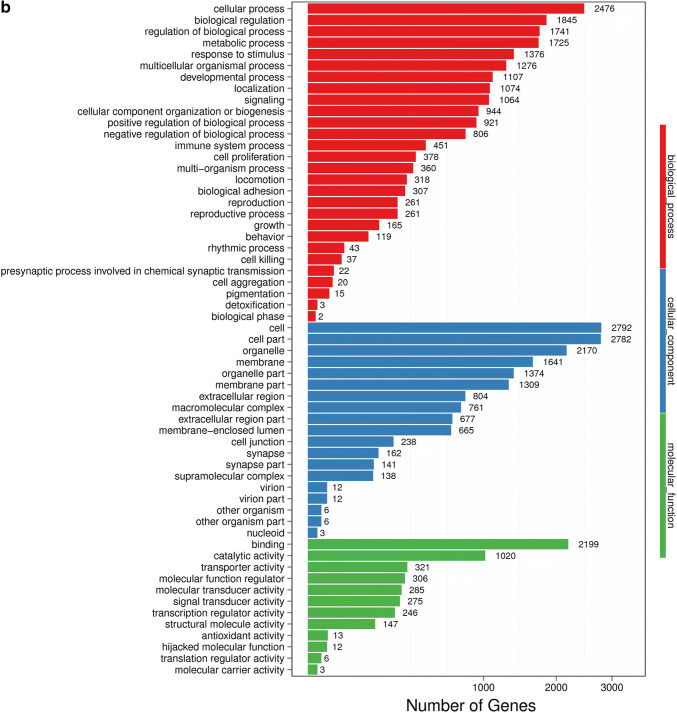

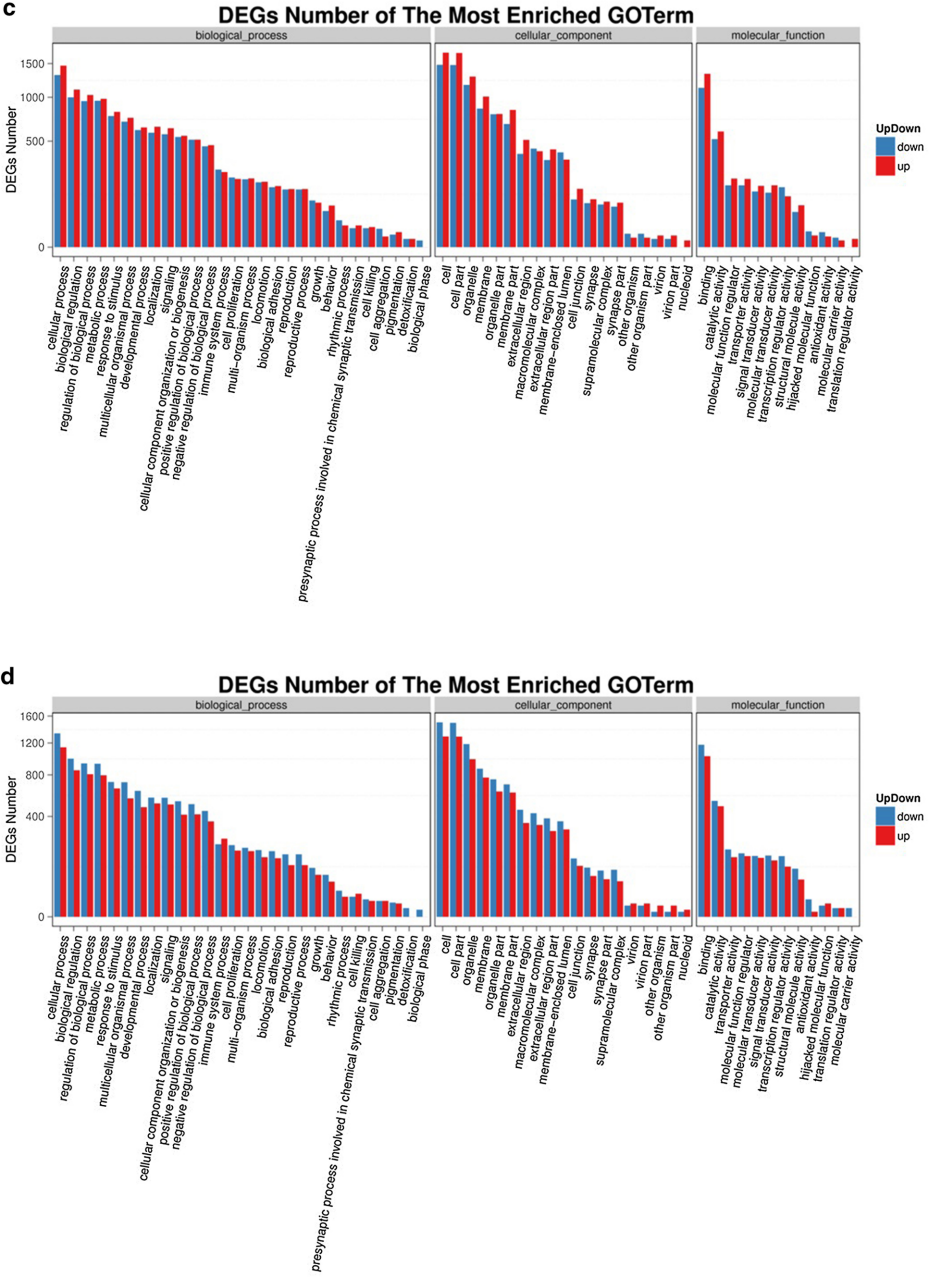
GO classification of DEGs. **a** NC vs sh1; **b** NC vs sh2. GO classification and functional enrichment among molecular biological functions, cellular components and biological processes. X axis represents the number of DEGs. Y axis represents GO terms. **c** NC vs sh1; **d** NC vs sh2. GO classification of upregulated and downregulated genes. X axis represents GO terms. Y axis represents the amount of up- or down-regulated genes

With DEGs, we performed Gene Ontology (GO) classification and functional enrichment. GO has three main ontologies: molecular biological function, cellular component and biological process. The GO classification results are shown in Fig. 2a, b. We used DAG (directed acyclic graph) to show the GO enrichment result. Each bar shows GO terms, and the amount of up- or down-regulated genes are shown in Fig. 2c, d. In our study, we found that TOX gene knockdown could significantly influence the cellular process, the cell growth as well as the death signal transduction, as was previously reported [7]. Among most of the enriched GO terms, most DEGs were related to cellular process, biological regulation and binding process.

### Pathway analysis of DEGs

**Fig. 3:**
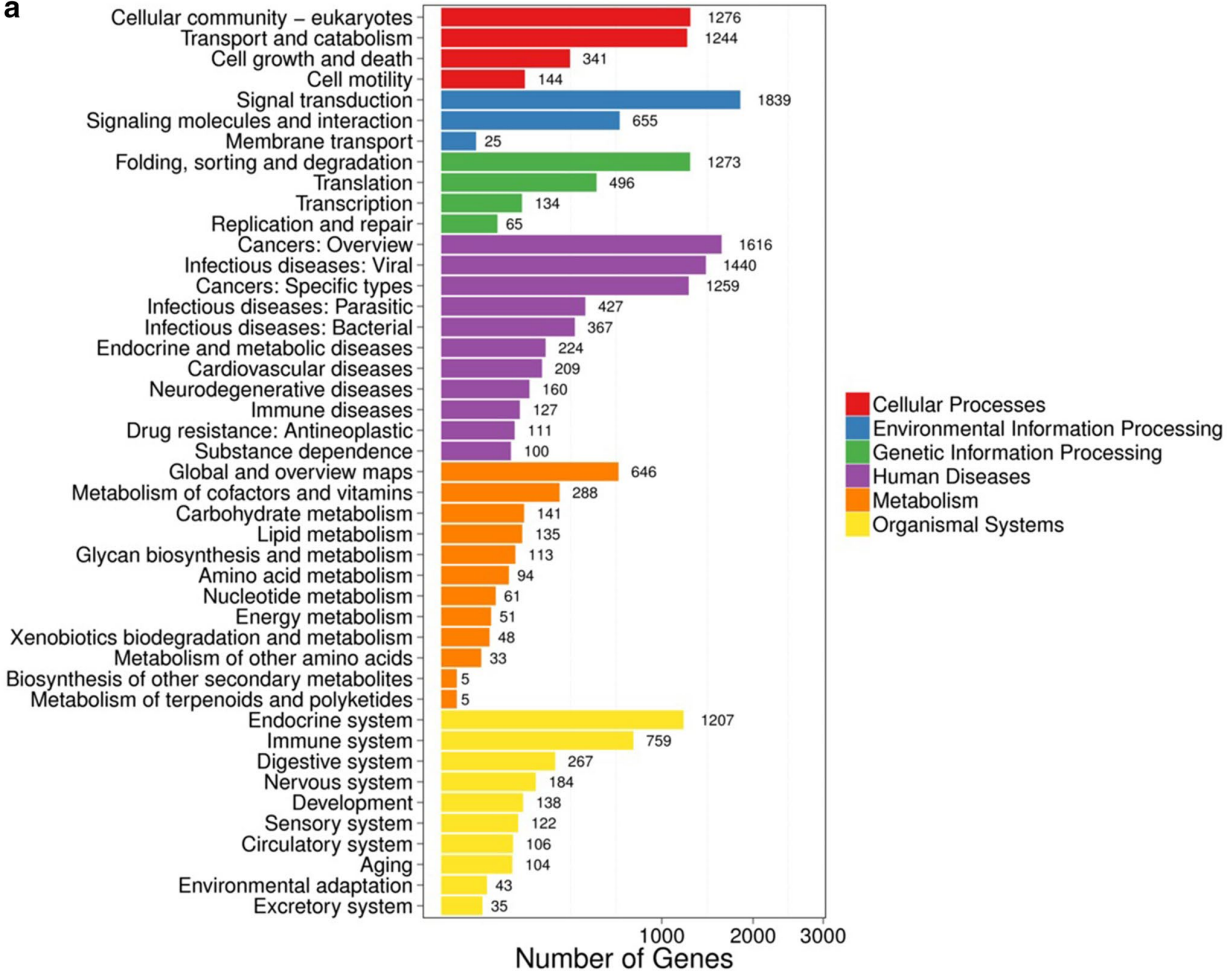

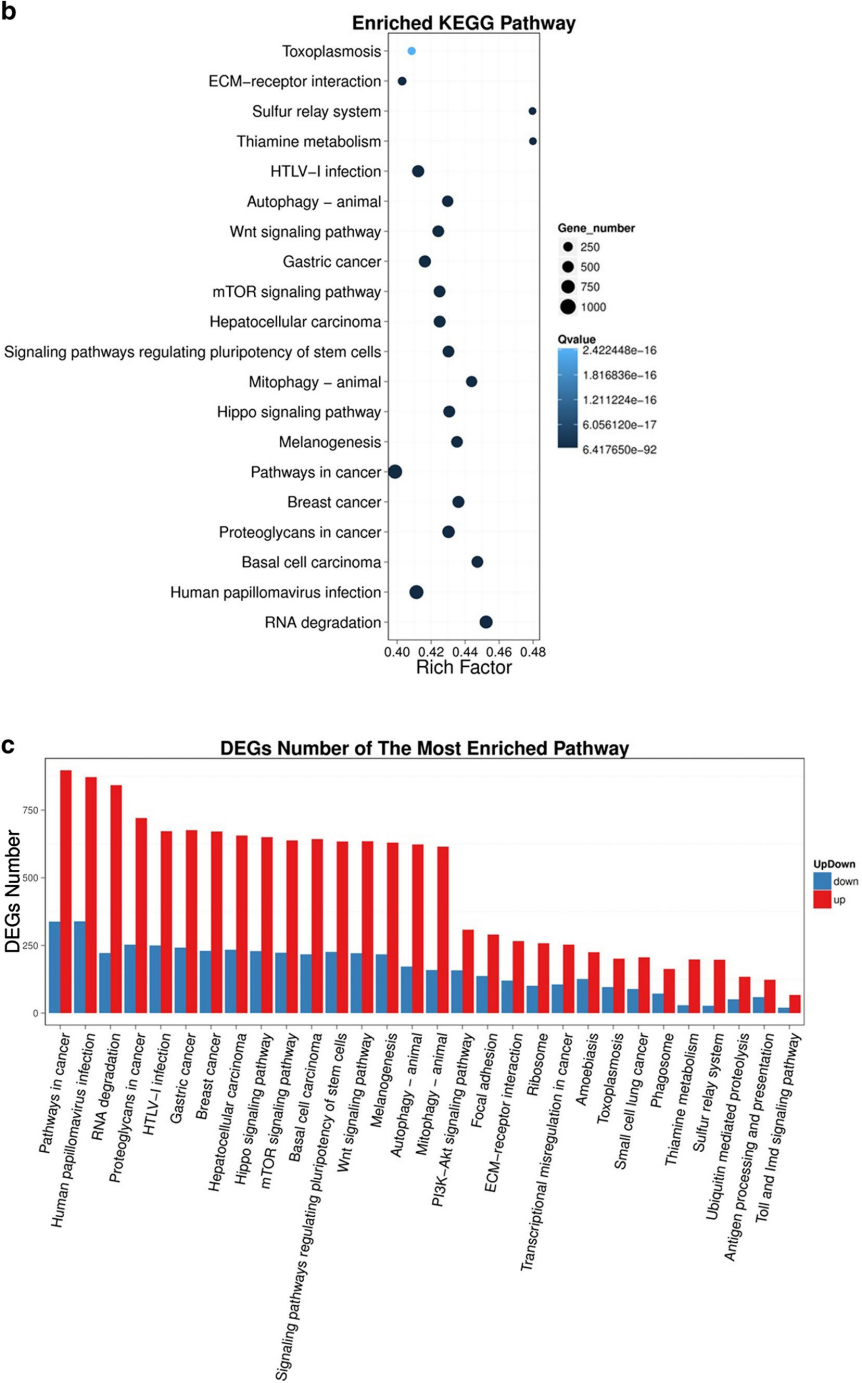
Pathway functional enrichment of DEGs between group NC and group sh1. **a** Pathway classification of DEGs; **b** Pathway functional enrichment of DEGs. **c** Pathway functional enrichment results for up- or down-regulated genes. X axis represents the term of pathways. Y axis represents the number of up- or down-regulated genes

To examine the expression profile of DEGs in our result, DEGs (both upregulated and downregulated) were then subjected to the KEGG pathway enrichment analysis. More than 23% of the DEGs could be annotated. The pathway classification results comparing group NC and group sh1/sh2 are shown in Fig. 3a and supplementary Fig. S2a, and the pathway functional enrichment results are shown in Fig. 3b and supplementary Fig. S2b. The pathway functional enrichment results for up- or down-regulated genes are shown in Fig. 3c and supplementary Fig. S2c. The top ten KEGG pathways with the highest representation of the DEGs are shown in Table 3. We found that most DEGs were enriched in cancer pathways (ko05200), including breast cancer (ko05224), gastric cancer (ko05226) and hepatocellular carcinoma (ko05225), and that some DEGs were also enriched in Wnt (ko04310), mTOR (ko04150) signaling pathways and pathways in regulating pluripotency of stem cells (ko04550).

**Table 3:** Top ten KEGG pathways with a high representation of DEGs

Pathway	DEGs genes with pathway annotation (5617)	All genes with pathway annotation (23,480)	*p* value	*q* value	Pathway ID
Pathways in cancer	1235 (19.8%)	3097 (13.19%)	8.962456e-68	4.959226e-66	ko05200
Melanogenesis	847 (13.58%)	1946 (8.29%)	3.684796e-64	1.595685e-62	ko04916
Wnt signaling pathway	856 (13.72%)	2018 (8.59%)	8.728826e-59	2.069979e-57	ko04310
Breast cancer	901 (14.44%)	2066 (8.8%)	4.650451e-69	3.087899e-67	ko05224
Gastric cancer	918 (14.72%)	2205 (9.39%)	8.712956e-59	2.069979e-57	ko05226
Proteoglycans in cancer	974 (15.61%)	2264 (9.64%)	2.275643e-71	1.888784e-69	ko05205
Hepatocellular carcinoma	890 (14.27%)	2094 (8.92%)	9.088525e-62	2.743082e-60	ko05225
Hippo signaling pathway	879 (14.09%)	2041 (8.69%)	3.845024e-64	1.595685e-62	ko04390
Signaling pathways regulating pluripotency of stem cells	860 (13.79%)	1999 (8.51%)	2.096168e-62	6.959278e-61	ko04550
mTOR signaling pathway	861 (13.8%)	2026 (8.63%)	1.376305e-59	3.807777e-58	ko04150

## Discussion

TOX is aberrantly overexpressed in CTCLs, such as MF and SS. Stable knockdown of TOX in CTCL cells reduces cell cycle progression and promotes apoptosis, leading to inhibited cell viability and colony-forming ability in vitro and suppressed tumor growth in vivo [12]. After TOX gene knockdown, many genes are highly expressed, such as two cyclin-dependent kinase inhibitors (CDKNs), including CDKN1B and CDKN1C) [15]. It has been reported that TOX is able to regulate cell cycle in primary Sézary cells and cutaneous T cell lymphoma, whereas TOX knockdown leads to cell cycle arrest and secondary cell death [12, 16]. In our study, we found that, after TOX knockdown, some proliferation and apoptosis-associated genes, such as PFKFB3, CDK5 and CKKN2A, were up- or down-regulated and most DEGs were enriched in cellular process and cancer pathways, which highlights the importance of TOX in cancer process. As we noted, both changes in apoptosis and cell cycle characteristics of the groups after gene knockdown, it is not clear if the differentially expressed genes are directly the result of interactions with TOX or the result of downstream cell cycle-dependent changes.

HOX genes, including HOXC9, HOXC4, HOXC5, HOXC8, HOXC10, HOXC11 and HOXC13, were significantly downregulated after TOX knockdown, with HOXC9 being downregulated to the highest degree. HOX genes are homeobox genes that function as transcription factors. In humans, 39 HOX genes have been assigned to 13 paralogous groups in four separate clusters termed HOXA, HOXB, HOXC and HOXD [8]. HOXC9 is aberrantly expressed in breast cancer, lung cancer, body fat mass and astrocytoma [3, 8, 14, 22]. HOXC9 can induce neuronal differentiation of neuroblastoma cells [26]. Wang et al. [25] demonstrate that HOXC9 can directly regulate distinct sets of genes to coordinate diverse cellular processes during neuronal differentiation. This may explain why TOX knockdown will lead to less cell viability and colony-forming ability in vitro and reduce tumor growth in vivo.

Through DEGs GO analysis, we found most DEGs are related to the cellular process, biological regulation and binding process. This can explain why TOX knockdown will induce inhibited cell viability as previously reported [7]. With DEGs pathway analysis applied in KEGG, we find two important tumor-related pathways, Wnt and mTOR. They are generally associated with cellular proliferation, differentiation and apoptosis in invertebrates and mammals [4]. β-catenin is expressed by tumor cells in cutaneous lymphoproliferative disorders at various frequencies, and activation and accumulation of β-catenin plays an important role in the development of skin lymphomas [1]. CTCL cells display mTORC1 activation in the lymphoma stage-related fashion with the highest percentage of positive cells identified at the late stage [17]. Treatment with rapamycin can persistently inhibit mTORC1 signaling, and the combined inhibition of mTORC1 and MNK could totally abrogate the growth of CTCL cells [18]. Taking together, these findings could help to understand the mechanism of action of TOX in CTCL and provide clues to novel therapeutics for CTCL.

Several strategies have been employed to enhance the efficacy of current treatments and to find new therapeutic options to improve survival and quality of life for patients with SS and other forms of advanced CTCL [19, 20, 30]. TOX encodes a high-mobility group family (HMG) domain binding nuclear protein which regulates the differentiation of developing T cells. It is thought of as a molecular marker for histological diagnosis of CTCL [6, 31]. Our work has addressed the role of DEGs after TOX knockdown, as GO functional enrichment and pathway analysis have indicated. A limitation of this work is that findings so far are restricted to a single cell line. However, we believe the results may provide some insights into the mechanism of TOX in CTCL as well as candidate targets for therapy of CTCL in the near future.

## Electronic supplementary material

Below is the link to the electronic supplementary material.

Supplementary file1 (PDF 784 kb)

Supplementary file2 (XLSX 25 kb)
