# Supplementary material for: Molecular profiling of TOX-deficient neoplastic cells in cutaneous T cell lymphoma
Source: Arch Dermatol Res. 2019 Nov 1;312(7):513–25. doi: 10.1007/s00403-019-02000-0 (PMC7354281; doi:10.1007/s00403-019-02000-0)
Supplement: Supplementary file 1 — Supplementary file1 (PDF 784 kb) [file 403_2019_2000_MOESM1_ESM.pdf]

Article title:

Molecular profiling of TOX-deficient neoplastic cells in cutaneous T cell lymphoma

Journal name:

Archives of Dermatological Research

Author names:

Jingkai Xu, He Huang, Shangshang Wang, Yanzhen Chen, Xueli Yin, Xuejun Zhang, Yaohua Zhang

Correspondence to:

Dr. Yaohua Zhang M.D. & Ph.D.

Affiliation:

[1] Institute of Dermatology, Huashan Hospital, Fudan University, Shanghai, China;

[2] Worldwide Medical Center, Huashan Hospital, Fudan University, Shanghai, China.

Email address: yvonne_zhang@ymail.com

Fig. S1 Different gene expression levels of various samples and groups.

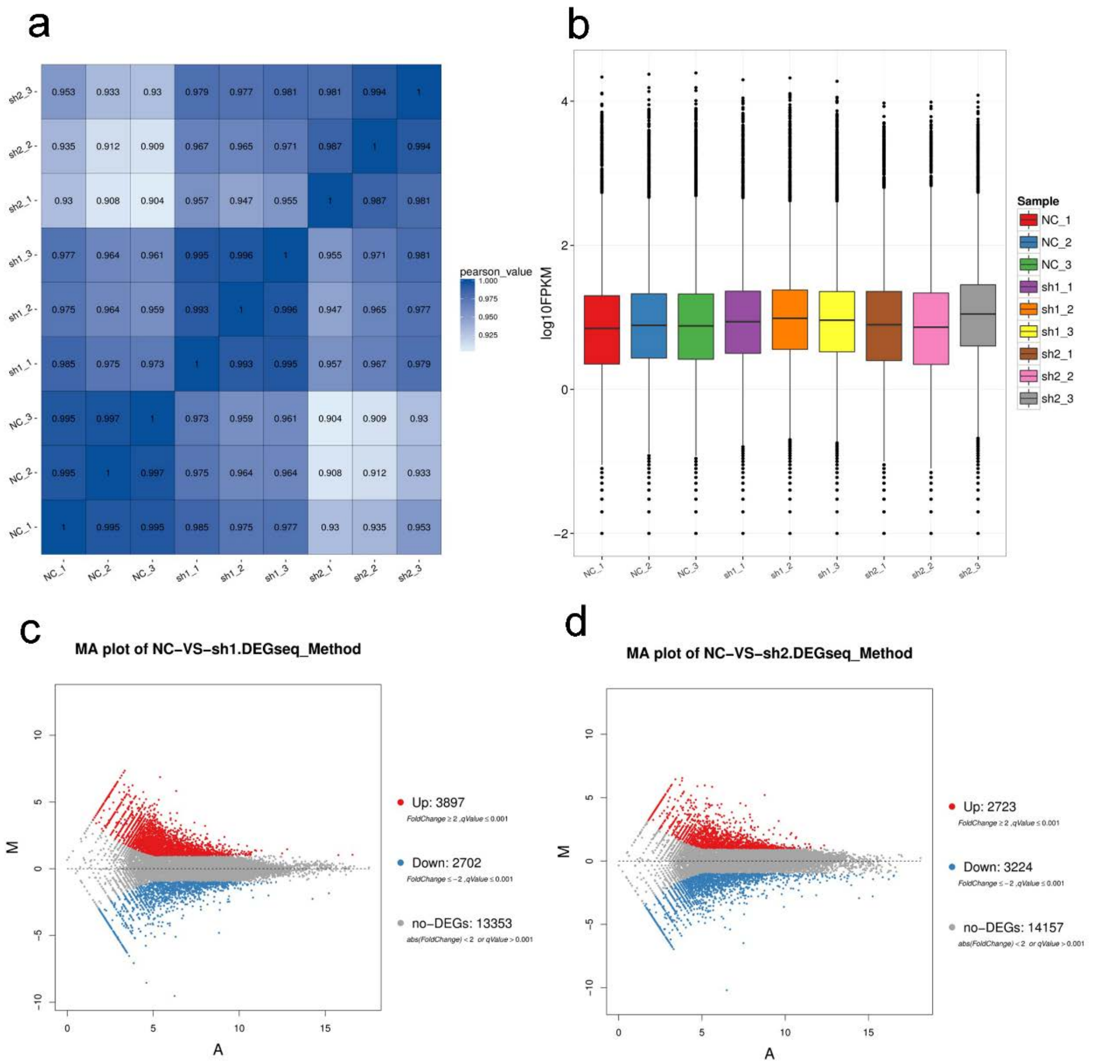

Note: a. Heatmap of Pearson correlation between samples. Both X and Y axes represent each sample. Coloring indicates Pearson correlation (high: blue; low: white). **b.** Gene expression Box-plot. X axis represents the samples. Y axis represents the log10FPKM value. **c.** Comparison results to NC group: 3897 genes over-expressed and 2702 genes down-expressed in sh1 group. **d.** Comparison results to NC group: 2723 genes over-expressed and 3224 genes down-expressed in sh2 group. X axis represents value A (log2 transformed mean expression level). Y axis represents value M (log2 transformed fold change). Red dots represent up-regulated DEGs. Blue dots represent down-regulated DEGs. Gray points represent non-DEGs.

Fig. S2 Pathway functional enrichment of DEGs between group NC and group sh2.

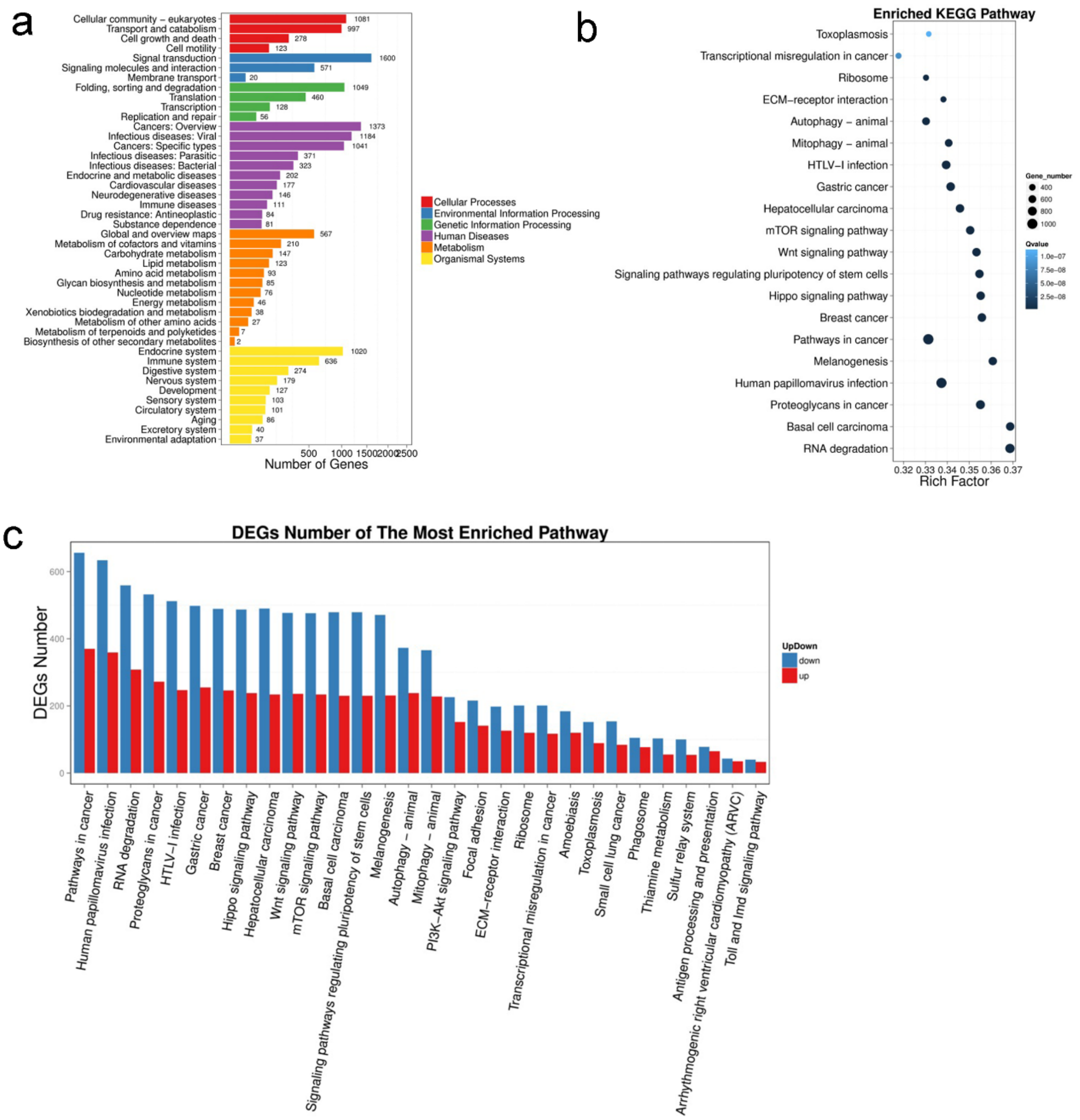

Note: a. Pathway classification of DEGs; **b.** Pathway functional enrichment of DEGs; **c.** Pathway functional enrichment results for up/down-regulated genes. X axis represents the term of Pathways. Y axis represents the number of up/down-regulated genes.

Table S1 Top 20 significantly up-regulated genes after TOX knockdown.

Gene Name	GeneID	Length[a]	NC-Expressio	sh1-Expressio	log2Ratio(sh1/NC)[c]	q-value[d]	p-value[e]	sh2-Expression	log2Ratio(sh2/NC)	q-value	p-value
**TGIF2-C20orf24**	100527943	1296	63.81	441.63	3.452196708	6.5453E-108	2.7519E-109	332.19	2.026921463	5.26E-33	2.41E-33
**ALPK1**	80216	5234	322	720	1.822149187	1.90395E-86	1.01479E-87	879	1.095569735	4.21E-35	1.75E-35
**GSTZ1**	2954	1395	153	448.27	2.212049268	1.39707E-69	1.09613E-70	530	1.439227965	1.43E-32	6.62E-33
**DEPDC1**	55635	5232	219	476	1.781243673	6.73847E-56	7.09388E-57	622	1.152750968	2.08E-27	1.22E-27
**BCL7B**	9275	1670	0	238.16	9.557000286	1.95665E-54	2.19588E-55	626.72	9.938444481	4.43E-93	4.96E-94
**PFKFB3**	5209	4080	453	700	1.289056841	3.33636E-51	4.15295E-52	1173	1.019387316	2.76E-41	9.04E-42
**ALKBH6**	84964	1074	252	491	1.62351226	9.54061E-51	1.2002E-51	814	1.338372319	4.27E-44	1.3E-44
**C7orf55-LUC7L2**	100996928	2747	352.64	592.68	1.410270215	8.42459E-50	1.10164E-50	1013.38	1.169674485	1.94E-44	5.84E-45
**RASA4B**	100271927	3136	8.87	154.88	4.787285912	1.23929E-46	1.81749E-47	233.36	4.364246635	5.67E-47	1.57E-47
**SPDYE6**	729597	2578	178.91	374.4	1.726559376	1.65377E-42	2.83048E-43	568.57	1.314871209	1.42E-30	7.17E-31
**IER3IP1**	51124	1504	377	566	1.247450499	1.01027E-39	1.93981E-40	1028	1.093971094	1.23E-40	4.11E-41
**ATP1A3**	478	1506	63.73	173.35	2.10485624	2.19773E-26	8.62887E-27	886.14	3.444257338	3.1E-149	2.1E-150
**RASA4B**	100271927	3136	8.87	154.88	4.787285912	1.23929E-46	1.81749E-47	233.36	4.364246635	5.67E-47	1.57E-47
**PAQR4**	124222	2370	253.83	436.09	1.441976287	2.67146E-38	5.54513E-39	1060	1.708897027	7.58E-81	1.01E-81
**CDK5**	1020	1202	160	319	1.656697488	1.66759E-34	4.05211E-35	568	1.474586283	5.6E-36	2.23E-36
**IER3**	8870	1254	69.08	205.91	2.236886877	2.83519E-33	7.31165E-34	377.83	2.098164527	7.04E-39	2.53E-39
**PSTPIP1**	9051	1940	153	338	1.804704563	7.10405E-41	1.2982E-41	509	1.380901261	1.43E-29	7.52E-30
**ILVBL**	10994	2185	237	359	1.260309754	2.92627E-26	1.15572E-26	910	1.587746744	7.51E-63	1.39E-63
**L1CAM**	3897	5126	0	64.19	7.665489625	2.0013E-19	1.3085E-19	253.12	8.630444953	1.75E-45	5.1E-46
**SYNJ2BP-COX16**	100529257	3832	0	155.71	8.943930759	4.87446E-39	9.74673E-40	174.26	8.091864896	1.11E-33	4.92E-34

Note: [a]Gene length; [b]gene expression of group NC; [c]log2 transformed fold change between NC group and sh1 group; [d]adjusted p-value; [e]p-val

Table S2. shRNA Target Sequence

Target name	Target Seq
**sh1**	ACTATCAGACTATTATCAA
**sh2**	ATGATACCTCTAAGATCAA
**NC**	TTCTCCGAACGTGTCACGT

Table S3. RT-qPCR primers

Gene ID	Primers Name	Primer Sequences	Products Length
**TOX**	TOX-F	ACATGGCCAGCTGACTACCA	269bp
	TOX-R	CAGGCTTCTGGGGCTCATTG	
**GAPDH**	qGAPDH-F	GATCATCAGCAATGCCTCCT	89bp
	qGAPDH-R	TGAGTCCTTCCACGATACCA	
